# Effect of intraoperative infusion of dexmedetomidine on postoperative recovery in patients undergoing endovascular interventional therapies: A prospective, randomized, controlled trial

**DOI:** 10.1002/brb3.1317

**Published:** 2019-05-17

**Authors:** Chunguang Ren, Huiying Xu, Guangjun Xu, Lei Liu, Guoying Liu, Zongwang Zhang, Jun-Li Cao

**Affiliations:** ^1^ Jiangsu Province Key Laboratory of Anesthesiology Xuzhou Medical University Xuzhou China; ^2^ Jiangsu Province Key Laboratory of Anesthesia and Analgesia Application Technology Xuzhou Medical University Xuzhou China; ^3^ Department of Anesthesiology Liaocheng People's Hospital Liaocheng Shandong China; ^4^ Department of Anesthesiology The Affiliated Hospital of Xuzhou Medical University Xuzhou China

**Keywords:** cerebral vasospasm, dexmedetomidine, endovascular interventional therapies, neuroanesthesia, nimodipine

## Abstract

**Background:**

Rapid emergence from general anesthesia during endovascular interventional therapies (EITs) is important. However, the solution that improved quality of both analepsia and postoperative recovery after EITs has not been specifically addressed. We conducted this prospective, randomized, controlled trial to evaluate the intraoperative infusion of dexmedetomidine on quality of analepsia and postoperative recovery in patients undergoing EITs.

**Methods:**

Eighty‐six patients undergoing EITs were divided into three groups: RD1 (dexmedetomidine at an initial dose of 0.5 μg/kg for 10 min adjusted to 0.2 μg kg^−1^ hr^−1^ throughout EIT), RD2 (dexmedetomidine at an initial dose of 0.5 μg/kg for 10 min adjusted to 0.4 μg kg^−1^ hr^−1^ throughout EIT), and RD3 (dexmedetomidine at an initial dose of 0.5 μg/kg for 10 min adjusted to 0.6 μg kg^−1^ hr^−1^ throughout EIT). An analgesia system delivered sufentanil only. The primary outcome measure was the total consumption of nimodipine during the first 48 hr after surgery. The secondary outcome measures were sufentanil consumption, pain intensity, hemodynamics, functional activity score (FAS), neurologic examination, level of sedation (LOS), and Bruggrmann comfort scale (BCS). We also recorded the intraoperative hemodynamic data, requirement of narcotic and vasoactive drugs, prevalence of complications and symptomatic cerebral vasospasm, duration of postanesthesia care unit (PACU) stay, Glasgow Outcome Score (GOS) at 3 months, and prevalence of cerebral infarction 30 days after surgery.

**Results:**

Dexmedetomidine application in the regimen RD3 reduced the consumption of the total dose of nimodipine and sufentanil 48 hr after surgery, prevalence of symptomatic cerebral vasospasm, consumption of narcotic drugs and nimodipine during surgery, pain intensity during the first 8 hr after surgery, and increased both BCS during the first 4 hr after surgery and hemodynamic stability. However, the LOS was increased at the 0.5 hr after surgery and surgeon satisfaction score was lower. There were no significant differences among the groups for consumption of vasoactive drugs except urapidil, Glasgow coma scale (GCS) and FAS during the first 48 hr after surgery, GOS at 3 months, and cerebral infarction after 30 days.

**Conclusions:**

Dexmedetomidine (an initial dose of 0.5 μg/kg for 10 min adjusted to 0.6 μg kg^−1^ hr^−1^ throughout EIT) could reduce the total consumption of nimodipine and opioid during the first 48 hr after surgery, the concerning adverse effects, and improve pain scores. The optimal dosage of dexmedetomidine during EITs merits further investigation.

## 1 INTRODUCTION

Patients with cerebrovascular diseases usually have considerable morbidity and (Gounis et al., 2015) in neuroanesthesia, the “ideal” state is that the brain is minimally affected by surgery and anesthesia and, simultaneously, autoregulation of the cerebral circulation is not damaged. Rapid recovery from neuroanesthesia and early neurologic examination are also important. Hemodynamic stability, especially with regard to arterial pressure to aid adequate cerebral perfusion, is a cornerstone of neuroanesthesia management (Flexman, Meng, & Gelb, 2015; Kundra, Mahendru, & Gupta, 2014). Studies have reported that 40%–80% of neurosurgical patients experience moderate‐to‐severe postoperative pain. Minimally invasive surgery is gaining popularity (Echegaray‐Benites, Kapoustina, & Gélinas, 2014; Goettel et al., 2016). Accordingly, several methods have been used for smooth emergence from general anesthesia during endovascular interventional therapies (EITs) (Berkhemer et al., 2016; Froehler et al., 2012; McDonagh et al., 2010).

Hemodynamic stability is important for minimizing extent of intracranial hemorrhage, as one of the most common causes of cerebral vasospasm (Griessenauer et al., 2017). Vasospasm can occur in 50% of patients undergoing EITs (Albanna et al., 2017; Levitt et al., 2014). Nearly one‐third of patients undergoing EITs arrive at intensive care units (ICUs) suffering from paroxysmal sympathetic hyperactivity (PSH), which can aggravate secondary brain injury. Several studies have focused on PSH treatment to prevent cerebral vasospasm after EITs, but a definitive solution is lacking (Baguley et al., 2014; Perkes, Baguley, Nott, & Menon, 2010).

Dexmedetomidine is a highly selective α2‐adrenergic agonist. It appears to have a partial neuroprotective effect in animal models of cerebral ischemia (Luo et al., 2017). Mechanisms presented for dexmedetomidine neuroprotective effect include α2A adrenoreceptor subtypes, brain‐derived neurotrophic factors, phosphoinositide 3‐kinase (P13K)/Akt, and extracellular signal‐regulated protein kinase (ERK)1/2 pathways (Wang et al., 2017). However, clinical application of dexmedetomidine alone or as an adjunct to remifentanil for EITs have not been reported adequately, though several studies have described usefulness of dexmedetomidine with or without remifentanil for craniotomy, none of the studies explored dexmedetomidine neuroprotective effects during and after EITs (Yun et al., 2017). We conducted this prospective, randomized, controlled trial to evaluate the effect of dexmedetomidine as an adjunct to remifentanil infusion in patients undergoing EITs.

## 2 MATERIALS AND METHODS

### 2.1 Ethical approval of the study protocol

Ethical approval of the protocol for this prospective, randomized, controlled clinical trial was obtained from the Institutional Review Board of Liaocheng People's Hospital (Liaocheng, China). Written informed consent for participation in this study was also obtained from all the patients or their guardian before participation in the study. The study was registered at chictr.org (ChiCTR‐IPR‐16008494).

### 2.2 Patients

Patients who underwent EITs from January 2017 to January 2019 were enrolled in this study if they met the following criteria: age 60–75 years; American Society of Anesthesiologists (ASA) grade I or II; diagnosed as having an unruptured cerebral aneurysm, arteriovenous malformation, or carotid artery stenosis using magnetic resonance imaging/angiography and three‐dimensional computed tomography (CT) angiography; having general anesthesia by tracheal intubation during surgery; transferred to the neurosurgical ICU; using a programmed syringe pump 48 hr after surgery.

Exclusion criteria were ruptured cerebral aneurysm or arteriovenous malformation; total intravenous anesthesia during surgery; admission planned to general wards; chronic renal failure (glomerular filtration rate < 30 ml/min); psychiatric disorders or receiving a psychotropic agent as medication; ischemic heart disease or second‐ or third‐degree heart block; alcohol, opioid, or sedative–hypnotic drug addiction; obesity (body mass index [BMI] > 30 kg/m^2^); to drugs used in the study; operative time <1 hr or >3 hr.

### 2.3 Randomization and masking

A computer‐generated randomization table was used to allocate the patients into three equal groups (*n* = 30 per group) by an independent anesthetist. After obtaining the patient's and their families' consent, the staff in the Acute Pain Services who was blinded to this study prepared the intravenous anesthetic agents and assessed pain intensity, the cumulative amount of self‐administered sufentanil and nimodipine, level of sedation (LOS), Bruggrmann comfort scale (BCS), functional activity score (FAS), and concerning adverse effects until 48 hr after surgery. Electronic charts and data from the DoCare clinic electronic anesthesia recording system were utilized. The anesthesia provider was blinded for patients assignment.

### 2.4 Anesthesia

Electrocardiography, arterial blood pressure, pulse oximetry, end‐tidal CO_2_, and temperature were monitored continuously using an automated system (IntelliVue MP50; Philips, Amsterdam, the Netherlands) after patients arrival at the operating room. A forced‐air warming device (EQ‐5000 Equator^®^ Convective Warmer; Minneapolis, MN) was used in both groups to maintain normothermia. Oxygen (100%) was administered via a facial mask at 4 L/min for 5 min. Dexmedetomidine was started at 0.5 μg/kg body weight for 10 min and then adjusted to 0.2–0.6 μg kg^−1^ hr^−1^ throughout the surgical procedure in the three groups. Fentanil (2–4 μg/kg), propofol (1–2 mg/kg), and cisatracurium (0.2 mg/kg) were administered via the intravenous route, and tracheal intubation was undertaken 3‐min later. Immediately after intubation, sevoflurane (1.5%–2.0%), remifentanil (0.05–0.15 μg kg^−1^ min^−1^), and nimodipine (5–20 μg kg^−1^ hr^−1^) were used for anesthesia maintenance. The pressure of arterial carbon dioxide (PaCO_2_) was maintained at 35–40 mmHg. Sevoflurane and dexmedetomidine were stopped 10 min before the end of the surgical procedure. The remifentanil infusion was continued until the femoral artery had been sutured. All patients received 4 mg of tropisetron and underwent routine reversal of neuromuscular blockade (atropine 4 μg/kg plus neostigmine 10 μg/kg). All surgical procedures were carried out by the same neurosurgeon.

Remifentanil infusion was adjusted by stepwise titration at 0.02 μg kg^−1^ min^−1^ according to acceptable hemodynamic limits (mean blood pressure [MBP] and heart rate [HR] maintained between ±20% of preoperative levels). For patients with a poor response to remifentanil, nimodipine was adjusted by stepwise titration at 5 μg kg^−1^ hr^−1^. Sevoflurane was adjusted by stepwise titration at 0.2% according to maintenance of the bispectral index (BIS) to 40–60 using a BIS monitor (Aspect Medical Systems, Newton, MA) as detailed in our previous reports (Shiyu, Ren, & Zhang, 2016). To elicit a satisfactory depth of anesthesia, vasoactive drugs were used intraoperatively to maintain hemodynamic stability. If necessary, phenylephrine (20–40 μg, i.v.) was administered intermittently or infused continuously at 10–30 μg kg^−1^ hr^−1^. Hypertension was treated with urapidil (10–15 mg, i.v.) tachycardia (HR > 100 beats/min) with control of sedation or esmolol 20 mg, atropine 0.2 mg was used at the time of HR <50 beats/min.

### 2.5 Postoperative management

Computed tomography of the brain was done in the catheter laboratory immediately after surgery to detect related acute complications such as hematoma or infarction (Zhang, Chen, Xiao, & Tang, 2017). Patients underwent complete neurologic examination by the same neurosurgeon after extubation, and then transferred to the postanesthesia care unit (PACU). The system of patient‐controlled anesthesia (sufentanil only) was programmed to deliver 2 ml/hr and 2 ml per demand with a 5‐min lockout interval, with a 1‐hr limit of 16 ml. Nimodipine (0.2–0.5 mg) was administered intermittently or infused at 5–20 μg kg^−1^ hr^−1^ to maintain systolic blood pressure (SBP) 110–120 mmHg after surgery (Zhang et al., 2017). Computed tomography or magnetic resonance angiography of the brain was done 6 hr after completion of the surgical procedure. If patients had neurologic symptoms and signs in the postoperative period, single‐photon emission CT of the brain was done to confirm the diagnosis of cerebral hyperperfusion syndrome (CHS).

### 2.6 Outcome measures

The primary outcome measure was the total consumption of nimodipine during the first 48 hr after surgery. Intraoperative hemodynamic data (MAP and HR) were obtained from the IntelVue monitor (Philips), at the following time points: arrival at the operating room (T1), before intubation (T2), intubation (T3); 5 min (T4), 10 min (T5), and 15 min (T6) after intubation; suturing of the femoral artery (T7); end of surgery (T8); extubation (T9); and 3 min (T10), 6 min (T11), 9 min (T12), 12 min (T13) after arrival at the PACU. Sufentanil consumption, pain intensity, hemodynamics, FAS as well as neurologic examination (Glasgow coma scale, GCS) were recorded at 1, 4, 8, 16, 24, and 48 hr after surgery. LOS was evaluated upon extubation as well as 0.5, 1, and 2 hr after surgery. Bruggrmann comfort scale was recorded at 1, 4, 8, 16, and 24 hr after surgery.

We also recorded the requirement of narcotic and vasoactive drugs, prevalence of complications and symptomatic cerebral vasospasm (the positive findings on transcranial Doppler (TCD) examination as a maximum flow velocity >200 cm/s or a mean flow velocity >120 cm/s at M1) (Ogami, Dofredo, Moheet, & Lahiri, 2017), duration of PACU stay and hospitalization, patients and surgeon satisfaction scores (on a 10‐point scale where 0 = poor, and 10 = excellent), (Berkhemer et al., 2016; McDonagh et al., 2010) GOS at 3 months, and prevalence of cerebral infarction 30 days after surgery.

### 2.7 Sample size

The sample size was calculated on the basis of an expected difference of 20% in the cumulative amount of nimodipine 48 hr after the surgery. For a study power of 80% (*α* = 0.05, *β* = 0.2), the required sample size per group was calculated to be 27, a total of 81 patients (PASS 11.0; ncss Statistical Software, Kaysville, UT). Assuming a dropout rate of 10%, the final sample size was determined to be 30 patients each group. Therefore, a sample size of 90 was chosen to allow for adequate data collection.

### 2.8 Statistical analyses

The Kolmogorov–Smirnov test was used to assess distribution of the variables. Homogeneity of variance was determined using Levene's tests. Normally distributed continuous variables were presented as the mean ± *SD* or inter‐quartile range. The Bonferroni's correction was used for post hoc multiple comparisons. Categorical data were expressed as frequencies and percentages and analyzed using chi‐squared tests or Fisher's exact tests if appropriate. *p* < 0.05 was considered significant. Statistical analyses were carried out using spss for Windows Version 16.0 (SPSS Inc., Chicago, IL).

## 3 RESULTS

### 3.1 Baseline characteristics

**Figure 1:**
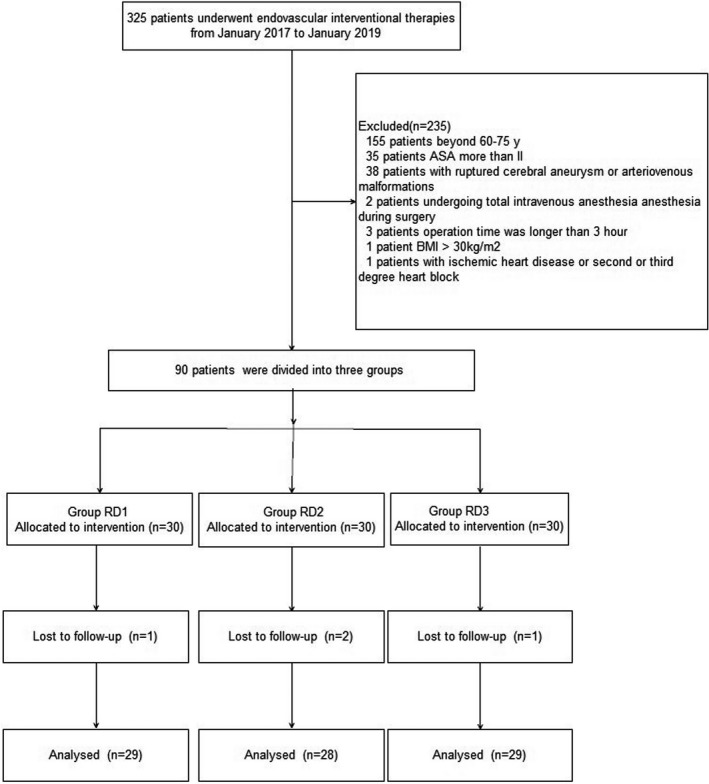
Patients enrollment flow diagram. This illustrates the flow of all patients screened, excluded, and randomized. ASA, American Society of Anesthesiology; BMI, body mass index

Consolidated standards of reporting trials diagram was used during the enrollment of patients (Figure 1). Three hundred and twenty‐five patients who underwent EITs from January 2017 to January 2019 were screened. Four patients were excluded as they were lost for follow‐up, and 86 patients were included in final data analysis (29 patients from group RD1, 28 patients from group RD2, and 29 patients from group RD3). Baseline characteristics and demographics of patients were not significantly different among the three groups (*p* > 0.05) (Table 1).

**Table 1:** Clinical characteristics of patients in the three groups

Variable	Group RD1 (*n* = 29)	Group RD2 (*n* = 28)	Group RD3 (*n* = 29)	*p*‐values
Age (years)	65.22 ± 3.42	66.25 ± 3.85	65.90 ± 3.30	0.797
Weight (kg)	65.66 ± 4.47	65.32 ± 4.74	67.52 ± 5.43	0.193
BMI (kg/m^2^)	23.24 ± 1.90	22.86 ± 2.70	22.62 ± 2.11	0.575
ASA I/II (*n*)	7/22	6/22	9/20	0.691
Sex (male/female)	12/17	15/13	17/12	0.402
Diagnosis, *n*（%）				0.943
Aneurysm	17 (58.62%)	15 (53.57%)	14 (48.28%)	
Arteriovenous malformation	2 (6.90%)	3 (10.71%)	3 (10.34%）	
Carotid artery stenosis	10 (34.48%)	10 (35.72%)	12 (41.38%)	
Comorbidity, *n* (%)				0.751
Hypertension	18 (62.07%)	13 (46.43%）	11 (37.93%)	
Diabetes mellitus	6 (20.69%)	4 (14.29%）	7 (24.14%)	
COPD/asthma	1 (3.45%)	2 (7.14%）	1 (3.45%)	
Coronary heart disease	2 (6.90%)	4 (14.29%)	4 (13.79%)	
GCS before surgery	14.25 (14.00–15.00)	14.50 (14.00–15.00)	14.50 (14.00–15.00)	0.732

Note

Variables presented as mean ± *SD*, median (interquartile range) or number of patients *n* (%).

Abbreviations: ASA, American Society of Anesthesiology; BMI, body mass index; COPD, chronic obstructive pulmonary disease; GCS, Glasgow Coma Scale.

### 3.2 Intraoperative variables

**Figure 2:**
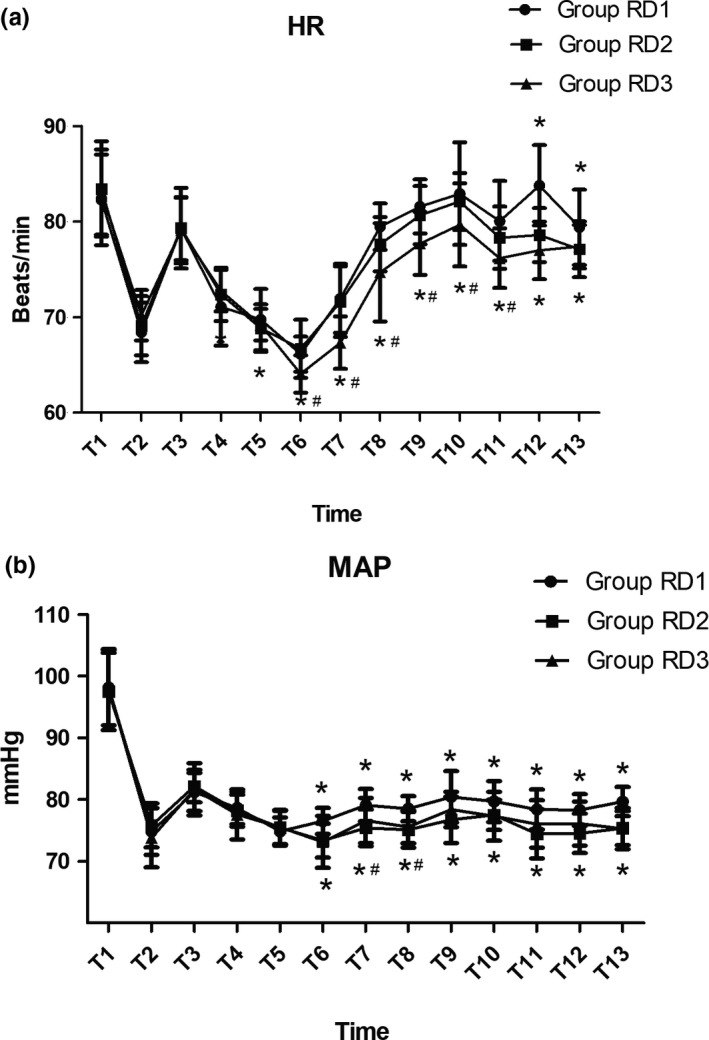
Hemodynamics was monitored in the three groups during the surgical procedure and postanesthesia care unit stay. (a) Comparison of the heart rate (HR; beats/min) in the three groups at different time points. (b) Comparison of mean arterial pressure (MAP; mmHg) in the three groups at different time points. Baseline vital signs were not significantly different between the two groups. *p < 0.05 versus Group RD1, ^#^ *p* < 0.05 versus Group RD2

Baseline vital signs were not significantly different among the three groups (*p* > 0.05) (Figure 2). Compared with the RD1 group, patients in the RD3 group showed significantly decreased HR from T5 to T13 (*p* < 0.05), while patients in the RD2 group showed significantly decreased HR only at T12 and T13 (*p* < 0.05). Compared with the RD2 group, patients in the RD3 group showed significantly decreased HR from T6 to T11 (*p* < 0.05) (Figure 2a). Patients in both RD2 and RD3 groups showed significantly decreased MAP from T6 to T13 (*p* < 0.05), while compared with the RD2 group, patients in the RD3 group showed significantly decreased MAP only at T7 and T8 (*p* < 0.05) (Figure 2b).

Compared with the RD1 group, the consumption of sevoflurane, remifentail, and nimodipine were significantly reduced in both RD2 and RD3 groups (Table 2). The consumption of dexmedetomidine was significantly increased (Table 2). Simultaneously, there was no significant difference among the three groups with regard to the duration of surgery and anesthesia, dose of propofol, cisatracurium, and fentanyl during surgery (Table 2). The dose of esmolol, phenylephrine, and urapidil were comparable among the three groups (Table 3). More patients in RD3 group need atropine during surgery (Table 3).

**Table 2:** Comparison of intraoperative variables in the three groups

Variable	Group RD1 (*n* = 29)	Group RD2 (*n* = 28)	Group RD3 (*n* = 29)	*p*‐values
Duration of surgery (min)	108.86 ± 12.57	107.21 ± 9.10	107.59 ± 11.68	0.729
Duration of anesthesia (min)	132.07 ± 12.99	130.00 ± 9.22	126.59 ± 10.89	0.175
Remifentanil dosage (μg)	865.68 ± 89.90	679.37 ± 70.48*	427.61 ± 53.17* ^,^ **	0.000
Dexmedetomidine dosage (μg)	59.50 ± 4.29	84.92 ± 7.49*	112.53 ± 12.30* ^,^ **	0.000
Nimodipine dosage (mg)	1.37 ± 0.14	1.06 ± 0.11*	0.78 ± 0.10* ^,^ **	0.000
Propofol dosage (mg)	117.59 ± 13.27	113.57 ± 15.92	114.14 ± 14.02	0.524
Cisatracurium dosage (mg)	20.48 ± 3.24	20.71 ± 3.18	20.07 ± 3.44	0.755
Fentanyl (mg)	0.23 ± 0.05	0.23 ± 0.04	0.21 ± 0.03	0.100
Sevoflurane (%)	1.73 ± 0.18	1.64 ± 0.13*	1.60 ± 0.16*	0.004

Note

Variables presented as mean ± *SD*.

* *p* < 0.05 versus Group RD1.

** *p* < 0.05 versus Group RD2.

**Table 3:** The consumption of vasoactive drugs during operation

Variable	Group RD1 (*n* = 29)	Group RD2 (*n* = 28)	Group RD3 (*n* = 29)	*p*‐values
Atropine	3 (10.34%)	2 (7.14%)	9 (31.03%)*	0.045
Esmolol	8 (17.24%)	3 (10.71%)	2 (6.90%)	0.106
Phenylephrine	6 (20.69%)	8 (28.57%)	8 (27.59%)	0.822
Urapidil	5 (17.24%)	3 (10.71%)	4 (13.79%)	0.779

Note

Variables presented as number of patients *n* (%).

* *p* < 0.05 versus Group RD2.

### 3.3 Postoperative variables

**Figure 3:**
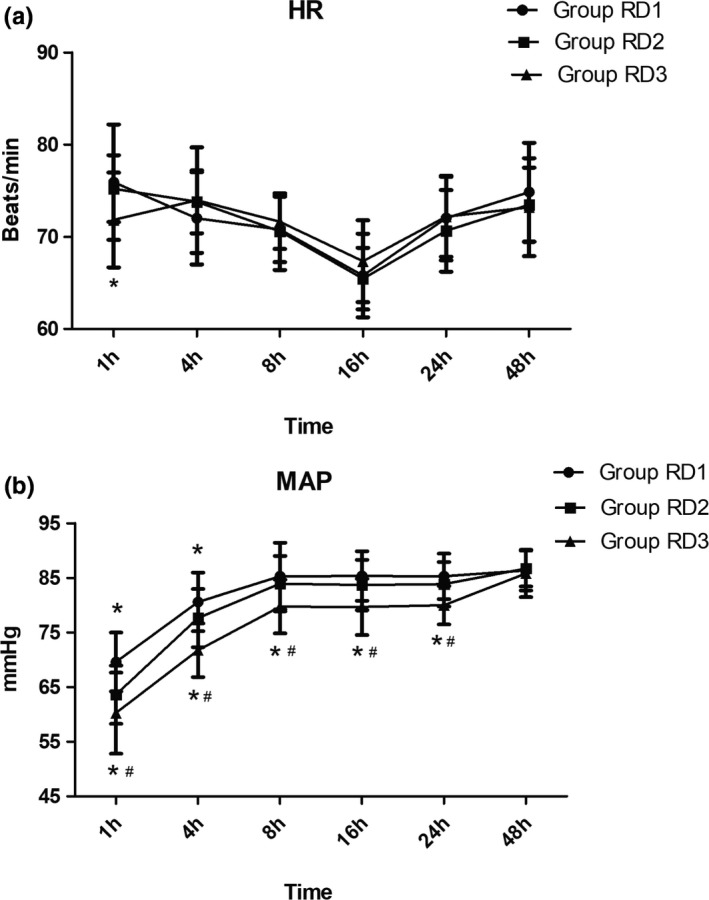
Hemodynamics was monitored in the three groups during the first 48 hr after surgery. (a) Comparison of heart rate (HR; beats/min) in the three groups at different time points. (b) Comparison of mean arterial pressure (MAP; mmHg) in the three groups at different time points. *p < 0.05 versus Group RD1, ^#^ *p* < 0.05 versus Group RD2

Compared with the RD1 group, patients in the RD3 group showed significantly decreased HR at 1 hr after surgery (*p* < 0.05) (Figure 3). Compared with the RD1 and RD2 groups, patients in the RD3 group showed significantly decreased MAP from 1 to 24 hr after surgery (*p* < 0.05). While compared with the RD1 group, patients in the RD2 group only showed significantly decreased MAP at 1 and 4 hr after surgery (*p* < 0.05) (Figure 3).

Intergroup comparison revealed that the recovery time in the PACU was significantly shorter for RD2 group (Table 4). The total dose of nimodipine was significantly lower in RD3 group 48 hr after surgery (Table 4). The number of applied urapidil was significantly higher in the RD1 group (Table 4). However, the surgeon's satisfaction score was higher in group RD1 compared with the other two groups, whereas the patient satisfaction score was comparable (Table 4). There were no significant differences among the three groups for duration of hospitalization, GOS of 3 months and cerebral infarction after 30 days (Table 4).

**Table 4:** The consumption of postoperative variables in the three groups

Variable	Group RD1 (*n* = 29)	Group RD2 (*n* = 28)	Group RD3(*n* = 29)	*p*‐values
Recovery time at PACU (min)	20.66 ± 6.34	17.21 ± 2.90*	19.59 ± 3.22**	0.015
Duration of hospitalization (day)	8.25 (6.25–14.50)	7.75 (6.25–13.75)	8.00 (7.00–13.50)	0.036
Nimodipine dosage (mg)	63.03 ± 4.30	43.90 ± 3.18*	22.69 ± 1.82* ^,^ **	0.000
Patient satisfaction score	7.28 ± 0.80	7.71 ± 0.96	8.00 ± 1.00	0.508
Surgeon satisfaction score	8.55 ± 0.63	8.26 ± 0.74	8.03 ± 0.82* ^,^ **	0.007
GOS of 3 months	4.00 (4.00–5.00)	4.00 (3.00–5.00)	4.00 (4.00–5.00)	0.564
Cerebral infarction after 30 days, *n*（%）	11 (37.93%)	9 (32.14%)	8 (27.59%)	0.703
Number of applied urapidil, *n* (%)	11 (37.93%)	3 (10.71%)*	3 (10.34%)*	0.012

Note

Variables presented as mean ± *SD*, median (interquartile range) or number of patients *n* (%).

Abbreviations: GOS, Glasgow outcome scale; PACU, postanesthesia care unit.

* *p* < 0.05 versus Group RD1.

** *p* < 0.05 versus Group RD2.

**Figure 4:**
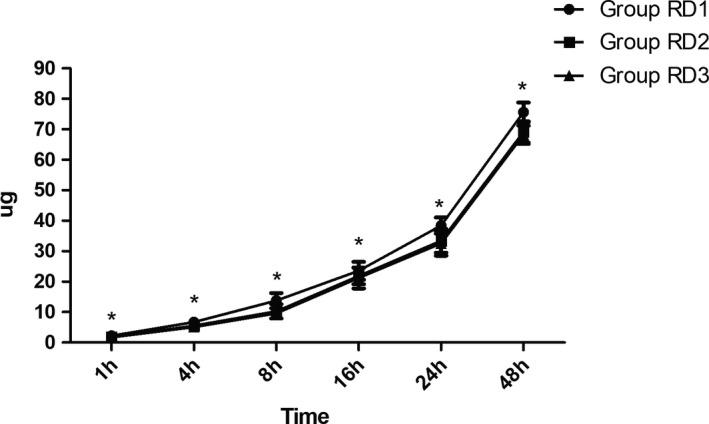
Postoperative consumption of sufentanil in the three groups. The total dose of sufentanil was significantly lower in RD3 group than the other two groups. *p < 0.05 versus group RD1

**Figure 5:**
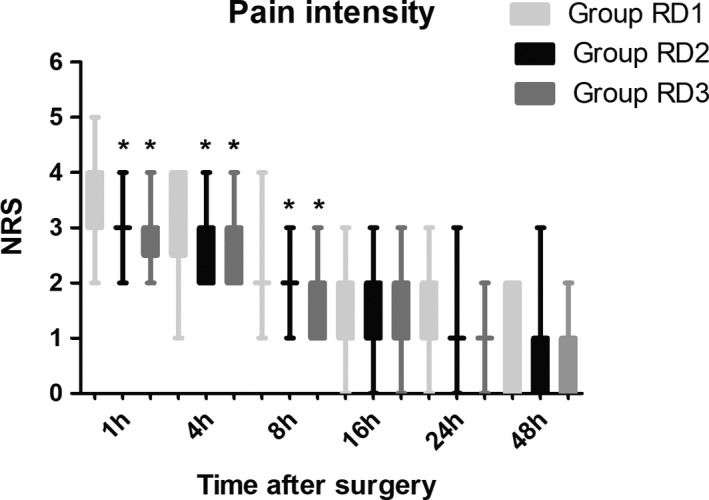
Time‐course of postoperative pain expressed as scores on a Numerical rating scale (NRS) out of 10 in the three groups. *p < 0.05 versus group RD

The total dose of sufentanil was significantly lower in RD3 group than the other two groups during the first 48 hr after surgery (Figure 4). Compared with RD1 group, the pain intensity was significantly lower in the other two groups at 1, 4, and 8 hr after surgery (Figure 5).

**Figure 6:**
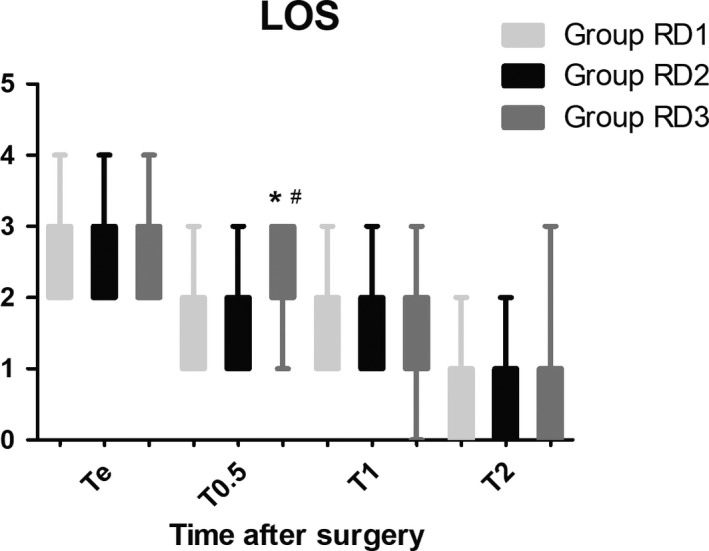
Comparison of patient sedation using the level of sedation (LOS) among the three groups. The LOS was only significantly higher in the RD3 group at 0.5 hr after surgery. Level of sedation: 1, subject is anxious, agitated, or restless; 2, subject is cooperative, oriented, tranquil and responds to commands; 3, subject is asleep but has a brisk response to light glabellar tap or a loud auditory stimulus; 4, subject is asleep, has a sluggish response to a light glabellar tap or loud auditory stimulus; and 5, subject is asleep and unresponsive. *p < 0.05 versus Group RD1, ^#^ *p* < 0.05 versus Group RD2

**Figure 7:**
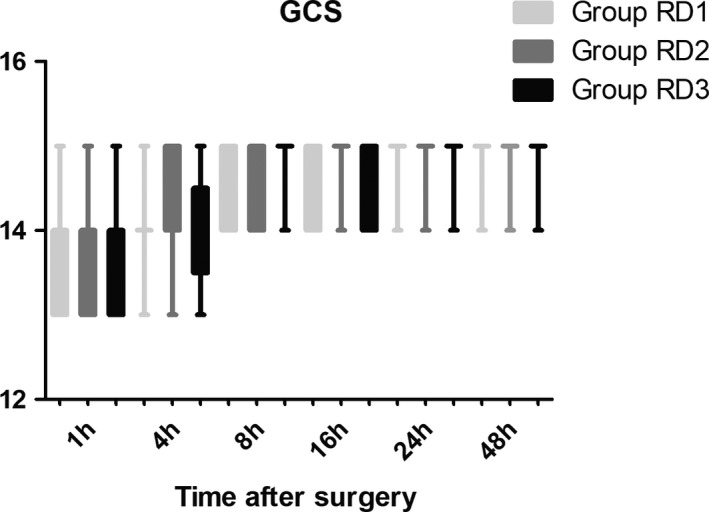
Comparison of Glasgow Coma Scale (GCS) scores among the three groups. There were no significant differences in terms of GCS during the first 48 hr after surgery

**Figure 8:**
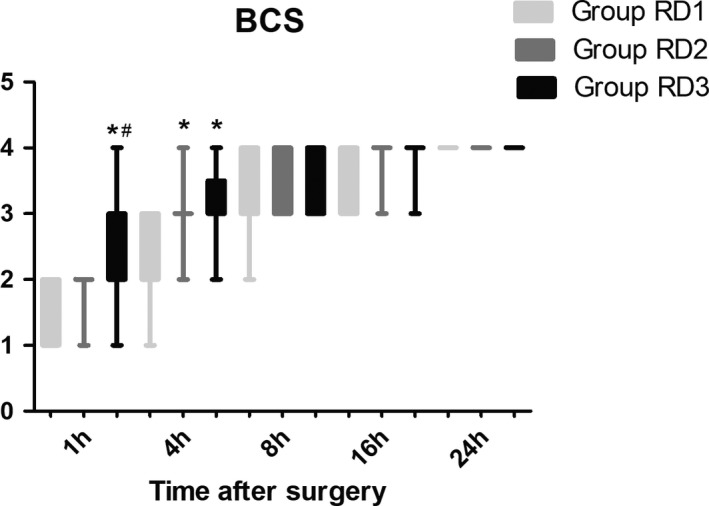
Comparison of Bruggrmann Comfort Scale (BCS) scores among the three groups. Bruggrmann Comfort Scale: 0, persistent pain; 1, severe pain while deep breathing or coughing; 2, mild pain while deep breathing or coughing; 3, painless while deep breathing; and 4, painless while coughing. *p < 0.05 versus Group RD1, ^#^ *p* < 0.05 versus Group RD2

The LOS was significantly lower only in the RD3 group 0.5 hr after surgery (Figure 6). There were no significant differences among the three groups in terms of the GCS and FAS during the first 48 hr after surgery (Figure 7 and Table 5). Compared with RD1 group, the BCS was significantly higher in RD3 group at 1 and 4 hr after surgery (*p* < 0.05) (Figure 8).

**Table 5:** FAS during 48 hr after surgery in the three groups

	Variable, hr	Group RD1 (*n* = 29)	Group RD2 (*n* = 28)	Group RD3(*n* = 29)	*p*‐values
FAS: C/B/A (*n*)	1	29/0/0	28/0/0	29/0/0	1.000
	4	29/0/0	28/0/0	29/0/0	1.000
	8	7/20/2	4/20/4	1/22/6	0.159
	16	0/21/8	0/20/8	0/14/15	0.126
	24	0/10/19	0/5/23	0/7/22	0.374
	48	0/0/29	0/0/28	0/0/29	1.000

Note

Variables presented as number of patients *n* (%).

Abbreviation: FAS, functional activity score (A, no restricted; B, mild‐to‐moderate restricted; and C, severely restricted).

The main adverse events were recorded (Table 6). Patients in RD1 group showed higher prevalence of symptomatic cerebral vasospasm (Table 6). There was no significant difference among the three groups with regard to the prevalence of nausea, tachycardia, bradycardia, hypotension, and hypertension (Table 6).

**Table 6:** Postoperative adverse events of patients in the three groups

Variable	Group RD1 (*n* = 29)	Group RD2 (*n* = 28)	Group RD3 (*n* = 29)	*p*‐values
Nausea	5 (17.24%)	5 (17.86%)	3 (10.34%)	0.751
Tachycardia	2 (6.90%)	2 (7.14%)	2 (6.90%)	1.000
Bradycardia	1 (3.45%)	1 (3.57%)	2 (6.90%)	1.000
Hypertension	7 (24.14%)	7 (25.00%)	8 (27.59%)	1.000
Hypotension	3 (10.34%)	2 (7.14%)	4 (13.79%)	0.905
Symptomatic cerebral vasospasm	12 (41.38%)	4 (14.29%)*	4 (13.79%)*	0.026

Note

Variables presented as number of patients *n* (%).

* *p* < 0.05 versus Group RD1.

## 4 DISCUSSION

In the prospective, double‐blind, randomized study, we found that dexmedetomidine at an initial dose of 0.5 μg/kg for 10 min, then adjust to 0.6 μg kg^−1^ hr^−1^ during surgery reduced consumption of the total dose of nimodipine and sufentanil 48 hr after surgery. More patients in the RD3 group need atropine during the surgery, which may be the principal reason for the lower surgeon satisfaction score found in the RD3 group. We also found that the consumption of sevoflurane, remifentail, dexmedetomidine, and nimodipine was significantly less in the RD3 group.

Compared with the RD1 group, patients in the RD2 and RD3 groups showed more stable hemodynamic profile. We found that the recovery time in the PACU was significantly shorter in the RD2 group. There were no significant differences among the three groups with regard to the GCS, BCS, or FAS during the first 48 hr after surgery, GOS at 3 months, or cerebral infarction after 30 days.

Endovascular interventional therapies are usually complicated procedures. Propofol, phenobarbital, dexmedetomidine, and benzodiazepine used alone or in combination with a short‐acting opioid such as remifentanil have been employed for monitored anesthesia care during EITs outside China (Amadori et al., 2013; Badenes, Gruenbaum, & Bilotta, 2015; Bustillo et al., 2002; McDonagh et al., 2010). Studies have reported that dexmedetomidine compared with local anesthesia could be considered to be a safe and effective method for cerebral angiography. It is associated with fewer hemodynamic fluctuations and postoperative complications and a shorter duration of hospital stay. However, patients are at risk of multiple complications, particularly respiratory depression (Banik & Prabhakar, 2015; Sriganesh, Reddy, Jena, Mittal, & Umamaheswara Rao, 2015). Hence, increasing number of patients and neurosurgeons prefer the use of general anesthesia during EITs, especially for complex surgical procedures.

Studies have shown that several types of adverse events can disturb cerebral oxygen delivery under general anesthesia, but no changes are observed with routine intraoperative monitoring methods (Kaku, Yamashita, Kokuzawa, Kanou, & Tsujimoto, 2012). Hence, general anesthesia is considered the first choice for EITs by most experts (Hoshino et al., 2010). Studies have also shown that the prevalence of cerebral vasospasm may be >50% during EITs, which can result in intracranial hypertension and cerebral ischemia. Eventually, cerebral vasospasm could increase the risk of postoperative complications and shorten the survival time of patients (Andereggen et al., 2017; Ogami et al., 2017). Hence, several methods have been adopted to reduce the risk of this complication. In our study, the rate of symptomatic cerebral vasospasm is lower in the RD3 group. However, the rate of cerebral infarction is the same in the three groups. The rate of cerebral infarction is just an imaging indicator, while symptomatic cerebral vasospasm is a comprehensive subjective indicators. There may be inconsistency between these two indicators. Previous studies have also addressed this problem (Gounis et al., 2015; Yun et al., 2017). Studies have also found that dexmedetomidine does not have a significant impact on cerebral perfusion or oxygen delivery in patients undergoing neurosurgery. However, the mechanism of action of dexmedetomidine on the control of intracranial pressure has not been clarified fully. Several factors may be involved, such as PaCO_2_, PaO_2_, temperature, pH, cerebral metabolic rate, and blood viscosity (Liu et al., 2018; Wenjie, Houqing, & Gengyun, 2014). Dexmedetomidine can decrease cerebral perfusion pressure, cerebral metabolic rate equivalent/cerebral blood flow ratio and increase cerebrovascular resistance. As a result, it can equilibrate the demand and supply of oxygen in the cerebrum, reduce excitotoxicity, and improve perfusion in the region of cerebral vasospasm (Ren, Ma, & Zuo, 2016; Sriganesh et al., 2015). Consistent with the previous results, we find that both the prevalence of symptomatic cerebral vasospasm and the total dose of nimodipine (the “gold standard” treatment of cerebral vasospasm) were higher in the RD1 group 48 hr after surgery, though the patient satisfaction score was similar among the three groups. These differences may have been due to the limitations of evaluation methods in our study. Besides, the surgeon's satisfaction score was higher in group RD1 compared with the other two groups. The reason maybe complex and partly because of too many interruptions during the surgery in the other two groups.

Less than 50% patients with uncontrolled blood pressure may suffer CHS. Though the total dose of nimodipine was significantly lower in group RD3 than the other two groups 48 hr after surgery, we did find a significant difference among the three groups with regard to the prevalence of symptomatic cerebral vasospasm. The reason may be due to the synergistic effect of nimodipine and dexmedetomidine though the specific mechanism has not been fully understood. These results are in accordance with a recent study which reported that intraoperative administration of dexmedetomidine could reduce the duration of CHS but not the prevalence of postoperative CHS in patients undergoing carotid endarterectomy (Suehiro et al., 2010). However, we did not find any significant difference among the three groups at both GOS of 3 months and cerebral infarction after 30 days.

Studies have demonstrated the neuroprotective property of dexmedetomidine. Also, studies have shown that dexmedetomidine can improve the microregional balance of the supply and consumption of oxygen by decreasing the heterogeneity of mixed venous oxygen saturation and the number of small veins with low oxygen saturation in an animal model of cerebral ischemia–reperfusion injury (Chi et al., 2015). Our studies have suggested that dexmedetomidine, compared with remifentanil, can reduce the dose of inhalation anesthetics and opioid drugs by about 30%(Shiyu et al., 2016). This effect was not obvious in the present study, which may be due to the different type of surgical procedure and drug dosage. However, this factor is very important for neurosurgical patients because use of inhalation anesthetics and infusion of opioid drugs after a long time can cause serious adverse events, such as postoperative delirium (Peng, Zhang, & Meng, 2017). Studies have reported that a single bolus and continuous intraoperative infusion of dexmedetomidine could result in fewer hemodynamic fluctuations than that for remifentanil infusion (Kim et al., 2016; Yun et al., 2017). We adopted the latter strategy for avoiding blood‐concentration fluctuations of dexmedetomidine and for investigating recovery and long‐term rehabilitation.

Endovascular interventional therapies for disorders of the nervous system are minimally invasive procedures. However, the prevalence of discomfort and pain after surgery can be ≤30%. Consistent with the results of a study by previous study, we also found that the total dose of sufentanil was significantly lower in RD3 group during the first 48 hr after surgery (Sriganesh et al., 2015). This synergistic effect may be because dexmedetomidine can act on α2A and α2C adrenoceptors in the spinal cord and brain, as well as modulating descending noradrenergic pathways to inhibit the release of glutamate from nerve terminals, suppression of voltage‐dependent Cav2.2 and Cav2.1 channels, and mitogen‐activated protein kinase activity (Jessen Lundorf, Korvenius Nedergaard, & Moller, 2016). This phenomenon is due to the unique pharmacologic properties of dexmedetomidine, which exerts its sedative effects through an endogenous sleep‐promoting pathway in the locus coeruleus of the cerebrum (Mueller et al., 2014). We found BCS was significant increased in the RD3 group during the first 4 hr after surgery. No patients need mechanical ventilation though LOS was significantly lower only in the RD3 group 0.5 hr after surgery.

Bradycardia and hypotension are the most commonly reported side effects associated with dexmedetomidine (Carollo, Nossaman, & Ramadhyani, 2008). We did record more patients in RD3 group need atropine during surgery than the other two groups (*p* = 0.045), most of which occurs at 15 min after intubation. However, there was no significant difference among the three groups with regard to the prevalence of nausea, tachycardia, bradycardia, hypotension, and hypertension after surgery. The reason may be due to the elimination half‐life of dexmedetomidine and duration of surgery (the prevalence of bradycardia after operation is mainly due to the usage of sufentanil). It has been demonstrated that delirium after EITs is an independent risk factor for greater neuropsychologic dysfunction and prolonged stay in hospital (Salata et al., 2012). Unfortunately, we did not record this indication in this prospective, randomized, controlled trial. However, dexmedetomidine has been employed to prevent delirium during ICU stay in several medical centers.

Our study had four main limitations. First, the number of patients was too small to make broad generalizations about dexmedetomidine use. Second, our study was a single‐center prospective, randomized, controlled trial. Third, plasma levels of catecholamines and dexmedetomidine were not assessed. Use of laser Doppler flowmetry and microdialysis could help to understand the action of dexmedetomidine at the tissue level. Finally, though TCD sonography is the most commonly used method for monitoring cerebral vasospasm in clinical research, we did not use it due to technical and economic reasons (Baumgartner, 2003).

## 5 CONCLUSIONS

We reported, for the first time, the application of dexmedetomidine (starting at 0.5 μg/kg for 10 min followed by adjustment to 0.6 μg kg^−1^ hr^−1^ throughout the surgical procedure) could reduce both the prevalence of symptomatic cerebral vasospasm and consumption of the total dose of nimodipine and sufentanil 48 hr after surgery, as well as increase the stability of hemodynamics. However, there were no significant differences among the three groups in terms of GCS and FAS during the first 48 hr after surgery, GOS at 3 months, or cerebral infarction after 30 days.

## CONFLICT OF INTEREST

The authors declared that the research was conducted in the absence of any commercial or financial relationships.

## AUTHOR CONTRIBUTIONS

Chunguang Ren, Jun‐Li Cao, and Zongwang Zhang conceived and designed the trial; Li Liu analyzed the data; Chunguang Ren, Huiying Xu, and Guangjun Xu collected the data; Chunguang Ren, Huiying Xu, Jun‐Li Cao, and Zongwang Zhang wrote this paper; Jun‐Li Cao and Zongwang Zhang contributed equally to this trail and should be considered co‐corresponding author.

## Data Availability

The data that support the findings of this study are available from the corresponding author upon reasonable request.
